# Mental effort and discomfort: Testing the peak-end effect during a cognitively demanding task

**DOI:** 10.1371/journal.pone.0191479

**Published:** 2018-02-12

**Authors:** Chia-Fen Hsu, Lee Propp, Larissa Panetta, Shane Martin, Stella Dentakos, Maggie E. Toplak, John D. Eastwood

**Affiliations:** 1 Department of Psychology, Faculty of Health, York University, Toronto, Canada; 2 Department of Psychology, Chung Shan Medical University, Taichung, Taiwan; 3 Clinical Psychological Room, Chung Shan Medical University Hospital, Taichung, Taiwan; Public Library of Science, UNITED KINGDOM

## Abstract

We applied the peak-end paradigm that was first introduced in the pain literature to examine the experience of effort and discomfort during a cognitively demanding working memory task. A total of 401 participants were asked to rate their effort and discomfort during and after the administration of a working memory task, which systematically varied task difficulty within participants and task duration between participants. Over the course of the task, participants reported a decrease in reported effort and an increase in reported discomfort. Peak and end real-time ratings were significant predictors of retrospective ratings for effort and discomfort; average and initial ratings predicted a small amount of additional variance. The regression analyses with effort and discomfort were largely consistent, with some exceptions. End discomfort significantly predicted willingness to do the task again, but not end effort. These findings highlight the ways in which the experience of effort and discomfort are integrally related, yet importantly separate, during a cognitively demanding task.

## Introduction

Sometimes thinking occurs relatively effortlessly, such as when we let our minds wander or when we complete an overlearned task. On other occasions controlling, corralling, and directing our conscious thought requires a great deal of mental effort. This phenomenological observation is relatively non-controversial. Notwithstanding the considerable debate regarding the definition and causal processes underlying the experience of mental effort [1–4], when considered from a functional perspective, there appears to be consensus that subjective mental effort might serve as a self-regulatory ‘signal’ that motivates a change in behaviour [5–8] or is taken as ‘input’ in decision-making processes [9–12]. In a nutshell, subjective mental effort serves a critical role in regulating our cognition, and it tends to be uncomfortable so we avoid tasks that require it [11–13].

The point of departure for the present work is the observation that different parts of an effortful event can be uniquely salient—these events have beginnings, middles and ends as well as relatively difficult and easy moments. Moreover, once completed, we form memories based on how uncomfortable we found the task and how much effort it required. Thus, it is important to understand the dynamics of subjective mental effort, and the possible association with discomfort, to understand how retrospective memories of cognitively demanding tasks are formed. These kinds of questions have been relatively well studied in the pain literature and that is where we turned for inspiration (e.g. [14]).

When asked to recall what it was like to undergo a painful procedure we do not simply sum up the total amount of pain experienced. Rather our memory appears to be primarily shaped by how painful it was at its worst moment and how painful it was when it ended. This has been called the ‘peak-end’ effect. Moreover, our memories of how painful the procedure was are not strongly impacted by the duration of the procedure, an observation that has been called ‘duration neglect’. In fact, we prefer to undergo a longer painful procedure as long as it ends less painfully [15]. We wondered if the peak-end and duration neglect effects observed during a painful procedure would also hold during the administration of a cognitively demanding task. Specifically, we examined real-time and retrospective ratings of effort and discomfort.

We are not the first to investigate whether peak-end and duration neglect effects apply to cognitively demanding tasks. Finn [16] has begun to address these questions (see also Hoogerheide & Paas [17]) and in the present study we extend these findings. Finn had participants compare two learning situations. In situation #1, participants had to learn a short list of difficult English-Spanish word pairs. In situation #2, participants had to learn an extended list of English-Spanish word pairs that was composed of the same number of equally difficult word pairs from situation #1 plus some additional, but easier to learn, word pairs that always occurred at the end of the task. Therefore, situation #2 was longer, and thus would have engendered greater total effort and discomfort. Nevertheless, participants preferred to repeat situation #2 and recalled it as being less difficult and uncomfortable than situation #1. These results suggest that how a cognitive task ends is critically important to retrospective memories and preferences for subsequent tasks. Recently, Finn & Miele [18] provided additional evidence showing that math tests that begin or end easier are more preferred than tasks which have easier items embedded in the middle. Taken together Finn’s work suggests that both easier beginnings and endings might play key roles in determining how mentally effortful tasks are remembered. Notably, unlike the pain research where ‘peak’ is defined by moments of most intense pain, Finn did not explore the impact of moments of most intense mental effort, but rather explored the impact of moments of relative ease.

We sought to probe similar questions, however, we employed a different experimental design and data analytic approach borrowed from the pain literature. Namely, we modelled our experimental design and data analytic approach on a study completed by Redelmeier and Kahneman [19]. During colonoscopy and lithotripsy surgeries, which were painful procedures with clear endings and beginnings, Redelmeier and Kahneman asked participants to report their level of pain every 60 seconds. Then, within one hour of completing the medical procedure participants were asked to recall the total amount of pain they experienced. Results indicated that participant’s retrospective judgments of total pain were strongly determined by the ratings of peak and end pain. Adding additional real-time pain ratings (such as initial levels, average level, and total) to the regression analyses provided a small, but significant, increase in the prediction of immediate retrospective judgments, and this increase in prediction appeared to be driven primarily by average real-time pain ratings. Importantly, however, the duration of the procedures was not related to retrospective judgments.

Similarly, we asked participants to report their level of discomfort and the amount of effort required of them every 60 seconds during a cognitively demanding task. Then, after the completion of the task, participants took a short break before retrospectively recalling their total amount of discomfort and effort. After the break we also asked them to retrospectively judge how well they thought they did on the task and how willing they would be to repeat the task again. We hypothesized that retrospective memory of how uncomfortable the task was would be predicted by peak and end levels of real-time discomfort, but not duration of the task. Moreover, we predicted that initial and average levels of real-time discomfort would add some additional predicative ability to retrospective memories, given this has also been found in the pain literature. We anticipated parallel findings for the prediction of retrospective memory of how much effort the task required.

Performance is an important characteristic of cognitively demanding tasks, thus we also examined whether perceived performance and actual performance would be predictors of retrospective discomfort, retrospective effort and willingness to repeat the task. Based on previous research showing that learners tracked, and remembered, their performance to make learning judgments [20, 21] and chose to study material that would maximize their future performance [22], we predicted that perceived performance would predict willingness to do the task again.

Modelling our experimental approach on the pain literature, we sought to extend previous work by Finn [16], Hoogerheide & Paas [17] and Finn and Miele [18]. In particular, we included five levels of task duration rather than only using short vs. long tasks. Furthermore, we manipulated duration independently from task difficulty and task performance. These design features allowed for a more direct test of ‘duration neglect’. We also measured real-time effort and real-time discomfort directly. Moreover, we used measures of real-time effort and real-time discomfort to predict retrospective memories of effort, discomfort, and willingness to repeat a cognitively demanding task. Therefore, we were able to directly explore and compare how various real-time experiences determine retrospective evaluations. Finally, in our design, peak moments of mental effort occurred independently of key temporal moments such as the beginning, and end of the task. Therefore, we were able to investigate the independent contribution of peak moments (un-confounded from beginnings and endings) in the prediction of retrospective memories of the task.

## Methods

### Ethics

The experimental protocol was reviewed and approved by a University Human Participants Review Committee before beginning data collection. The study was conducted according to the principles expressed in the Declaration of Helsinki. Informed consent was obtained from the participants.

### Participants

A total of 401 participants were included in the final analyses of the present study (mean age: 20.59, SD: 4.97, age range: 17 to 55, female: 68%). Participants were recruited from a university in a Canadian city. The inclusion criterion was being a fluent English speaker for the past ten years. Three consecutive academic semesters were required to recruit all of the participants. Course credit was given in exchange for participation. The total sample consisted of 431 participants. However, the data of 30 participants were excluded from the analyses, as follows: eight participants were deemed to be outliers due to extreme performance on the working memory task (i.e. accuracy at any block was more than three standard deviations away from the mean of the blocks with the same difficulty), 10 participants were dropped because they failed the practice trials on the working memory task, five participants had missing data or a technical failure during administration, four participants failed to follow instructions and three participants reported having ADHD. Given the novelty of our research questions and experimental design there was no way for us to estimate effect sizes. Thus, we decided to collect data from a relatively large number of participants so as to arrive at more stable findings and better estimates of effect sizes. Specifically, we decided a priori to collect as many participants as we could from our undergraduate research pool across three consecutive academic terms (Winter, Summer and Fall) with the understanding that approximately 150 participants would be available to us in the winter and fall and approximately 100 would be available in the spring.

### Working memory task

**Fig 1:**
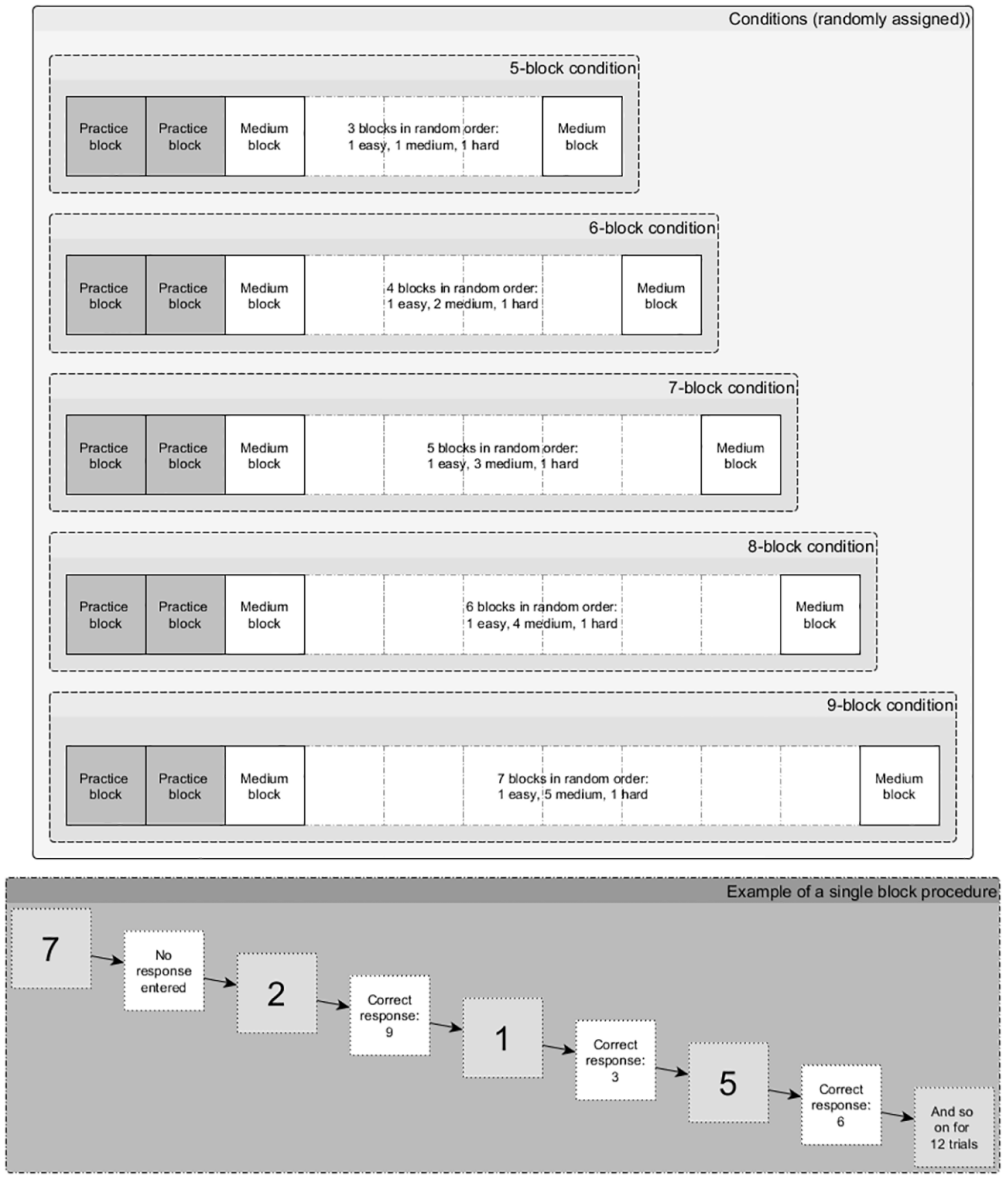
The modified Paced Auditory Serial Addition Test.

We modified the Paced Auditory Serial Addition Test (PASAT, [23]) to induce the experience of mental effort. Participants were asked to continuously add numerical digits presented visually on a computer monitor. Each digit was presented on the screen for one second. An orienting auditory beep was simultaneously presented with each digit. The delay between the offset of one digit and the onset of the next digit was 4 seconds. Participants were instructed to sum the last two digits they had seen and to enter their response using a keyboard. Participants were able to respond as soon as the latest digit appeared and during the 4 second delay; thus, within a response window of 5 seconds. Participants were not given feedback on their performance. Each ‘block’ required participants to provide 15 sums (i.e. 15 trials with a total of 16 digits presented). The task had five conditions differing in duration, i.e. the number of blocks ranged from five to nine blocks. Participants were randomly assigned to one of five conditions. Each block was one of three levels of difficulty: hard, medium or easy, which were manipulated within participants. The hard trials involved adding one single and one double digit. The medium trials involved adding two single digits that summed to more than nine. The easy trials involved adding two single digits that summed to nine or less. The blocks were presented in a pseudo-randomized sequence, as the first and last blocks were always medium difficulty. The middle blocks were composed of one easy, one hard and one to five medium blocks (depending on the duration condition administered, see Fig 1).

Before the task, participants had to complete two practice blocks (medium difficulty) to ensure they understood the task. Before completing the practice blocks participants were asked to complete a number of self-report scales of trait individual differences. After completing the two practice blocks participants were asked to anticipate how much effort the task would require and how much discomfort the task would engender. Results from the trait measures and the anticipation questions are not examined in the present manuscript. During the task participants were prompted to answer two questions after each block (every 15 responses, or once per minute) about their experience of effort and discomfort. The questions were (1) “Rate your current level of mental effort” (1 = None; 7 = A lot) and (2) “Rate your current level of discomfort or distress” (1 = None; 7 = A lot). From these ratings we calculated the peak (maximum rating), end (rating of the final block), average, initial (rating of the first block), and total (area under curve) ratings of effort and discomfort, as well as the slope of the linear trend in change of real-time effort and discomfort (average of all participants with each participant’s slope calculated based on the particular duration they underwent). After they finished the task, participants proceeded to complete demographic information, which created a 3 to 6 minute time lag, before they retrospectively evaluated their experience. The retrospective questions included (1) “On this working memory task, what was your total amount of mental effort?” (1 = None; 7 = A lot); (2) “On this working memory task, what was your total amount of discomfort or distress?” (1 = None; 7 = A lot); (3) “How well did you perform on the working memory task?” (1 = Significantly below average; 7 = Significantly Above Average); and (4) “How willing would you be to do another working memory task right now?” (1 = Not at all willing; 7 = Definitely willing). These ratings represented participants’ retrospective discomfort, effort, perceived performance and the willingness to repeat task respectively.

Participants were recruited for three consecutive semesters using a similar procedure of the PASAT and the testing was conducted in the same location. However, in the first two semesters, the PASAT was programmed using E-prime [24] while questions of demographic and retrospective evaluations were presented separately on Qualtrics survey software (Qualtrics, Provo, UT). In the third semester, the PASAT and retrospective questions were programmed using jsPsych [25]. The jsPsych program applied a passing standard for the second practice block, instead of relying on experimenters to track and provide feedback at the outset of the experiment. In the jsPsych version of PASAT, participants had up to three chances to repeat the practice block. Participants whose performance was lower than 80% after three attempts did not complete the study. In both instantiations of the experimental procedure participants were not given feedback on their performance once the practice blocks were completed. The data patterns obtained from these three sessions yielded consistent findings.

### Procedure

All materials were presented to participants individually and recorded using a desktop computer within a single session. Participants completed the working memory task followed by demographic questions. Subsequently participants completed retrospective evaluations about the working memory task. The total duration of the experiment ranged from approximate 40 to 50 minutes.

### Statistical analyses

Data were analysed using the SPSS version 22 statistics software package. Descriptive data of real-time effort and discomfort ratings were reported. The linear trend of real-time effort and discomfort over time was examined using one sample t-test and the difference between the linear trend of real-time effort and discomfort was tested using paired t-test. One-way repeated measure ANOVAs were applied to compare the differences in real-time effort, discomfort and accuracy among blocks with different levels of difficulty. Pearson’s correlation coefficients were computed among real-time ratings of effort and discomfort, retrospective evaluations and accuracy. Hierarchical regressions were conducted to evaluate the adequacy of peak and end ratings in the prediction of retrospective evaluations and the willingness to repeat task.

## Results

Participants generally found the PASAT to be effortful and uncomfortable. Thirty-eight percent of participants reported a maximum effort score of 7 at least once during the task and twenty-eight percent of participants reported a maximum discomfort score of 7 at least once during the task. Moreover, we investigated the linear trend of real-time effort and discomfort over time by calculating the linear trend of each participant (i.e. calculated based on the particular duration that participant underwent) and then averaging these slopes to create a group mean. Our results indicate that effort and discomfort changed significantly over time. Namely, as the task went on participants found that it became less effortful (mean slope = -.04; and differed significantly from zero, *t*(400) = -2.70, *p* = .007) and more uncomfortable (mean slope = .04; and differed significantly from zero, *t*(400) = 2.95, *p* = .003). Moreover, the slope of real-time effort was significantly different from the slope of real-time discomfort (*t*(400) = -5.51, *p* <.001). See Table 1 for other descriptive statistics.

**Table 1:** Mean (SD) ratings, trends and performance on the PASAT.

	Mean	SD	(Min, Max)
Real-time ratings			
Peak discomfort	5.02	1.82	(1,7)
End discomfort	3.90	1.88	(1, 7)
Average discomfort	3.84	1.57	(1, 7)
Initial discomfort	3.58	1.71	(1, 7)
Total discomfort	22.86	11.01	(4, 51)
Peak effort	5.85	1.19	(2,7)
End effort	4.49	1.66	(1, 7)
Average effort	4.68	1.27	(1.2, 7)
Initial effort	4.73	1.41	(1, 7)
Total effort	27.64	9.61	(5, 56)
Linear trend in discomfort	.04	.30	(-1.5, 1.2)
Linear trend in effort	-.04	.28	(-1.09, 1.11)
Retrospective ratings			
Retrospective discomfort	4.17	1.7	(1, 7)
Retrospective effort	5.09	1.37	(1, 7)
Perceived performance	4.07	1.35	(1, 7)
Willingness to do the task again	3.55	1.98	(1, 7)
Performance accuracy	.76	.15	(.3, 1)

Average mental effort and average discomfort were significantly correlated (*r* = .61; *p*< .001). The mean slope of real-time effort and the mean slope of real-time discomfort were significantly correlated (*r* = .48; *p* <.001). Thus, a robust relationship was observed between effort and discomfort, confirming the generally accepted idea that engaging mental effort is aversive.

Table 2 shows means and standard deviations for real-time effort, discomfort and accuracy during the easy, medium and hard blocks of the working memory task. One-way repeated measure ANOVAs indicated that real-time effort, discomfort and accuracy all showed a strong main effect of task difficulty (*p* < .001). The post hoc analysis indicated the hard, medium and easy conditions differed significantly (all *p* < .001). That is, difficult blocks were rated as more effortful and more uncomfortable, and were completed with less accuracy than medium and easy blocks; medium and easy blocks also differed in the same manner. These findings confirm our manipulation of task difficulty was successful.

**Table 2:** Mean (SD) for accuracy, real-time ratings of effort and real-time ratings of discomfort across levels of task difficulty.

	Easy	Medium	Hard	F (2, 399)
Accuracy	0.82 (0.18)	0.77 (0.16)	0.60 (0.24)	177.76***
Real-time effort	4.27 (1.65)	4.59 (1.31)	5.49 (1.44)	144.10***
Real-time discomfort	3.40 (1.79)	3.78 (1.59)	4.59 (1.89)	127.24***

***p <.001

Mirroring Redelmeier and Kahneman’s [19] findings for pain, peak and end ratings of real-time discomfort were strongly correlated with retrospective discomfort (peak real-time discomfort and retrospective discomfort: *r* = .84, *p* <.001; end real-time discomfort and retrospective discomfort: *r* = .76, *p* <.001). Furthermore, peak and end levels of real-time discomfort were correlated with willingness to do the task again (peak discomfort and willingness to do the task again: *r* = -.19, *p* = .001; end discomfort and willingness to do the task again: *r* = -.24, *p* <.001).

Peak and end ratings of real-time effort were strongly correlated with retrospective effort (end effort and retrospective effort: *r* = .66, *p* <.001; peak effort and retrospective effort: *r* = .71, *p* <.001). However, peak and end levels of real-time effort were not correlated with willingness to do the task again (end and willingness to do the task again: *r* = -.08, *ns*; peak and willingness to do the task again: *r* = -.07, *ns*). There was no significant correlation between task duration and retrospective discomfort (*r* = -.03, *ns*) or between task duration and willingness to do the task again (*r* = -.03, *ns*) or between task duration and retrospective effort (*r* = -.03, *ns*). Correlations between all variables are displayed in Table 3.

**Table 3:** Correlations between effort, discomfort, perceived performance, willingness to do task again and accuracy.

	Retrospective effort	Retrospective discomfort	Perceived performance	Willingness to do task again	Accuracy
Peak effort	.71***	.54***	-.14**	-.07	-.24***
End effort	.66***	.48***	-.19***	-.08	-.26***
Average effort	.77***	.57***	-.15**	-.09	-.25***
Initial effort	.58***	.43***	-.08	-.05	-.17**
Total effort	.58***	.44***	-.09	-.09	-.13**
Peak discomfort	.54***	.84***	-.26***	-.19***	-.30***
End discomfort	.50***	.76***	-.29***	-.24***	-.32***
Average discomfort	.55***	.86***	-.28***	-.23***	-.31***
Initial discomfort	.46***	.70***	-.23***	-.16**	-.25***
Total discomfort	.46***	.72***	-.22***	-.20***	-.21***
Duration	-.03	-.03	.05	-.03	.11*

***p <.001;

**p <.01;

*p <.05

In a manner similar to Redelmeier and Kahneman [19], two separate stepwise regressions were conducted in order to evaluate the adequacy of peak and end discomfort in the prediction of retrospective evaluations. Two parallel regression analyses were conducted with the real-time effort ratings given the strong relationships observed between real-time effort and discomfort. Peak and end real-time ratings were entered in the first step and this served as a reference point for comparing several other more complex models, which included additional predictors. More complex models were compared to the simple model serially so as to minimize any potential problems with multicollinearity. We examined the variance inflation factor (VIF) to detect any problems with multicollinearity in our analyses. Across the regression analyses, the VIF scores ranged from 1.0 to 1.8, with the exception when average effort and discomfort were entered in a separate block and the VIF score was 5.64 and 7.03, respectively; these scores are below the recommended cut-off of 10 for VIF scores and also acceptable given our a priori and theoretically motivated rationale to examine whether peak, end and average ratings would be unique predictors in our regression analyses [26]. Given the number of statistical tests conducted we employed a Bonferroni correction to significance testing (α = .003). The results were presented in Tables 4 and 5.

**Table 4:** Hierarchical regression analysis predicting retrospective discomfort and the willingness to do the task again from real-time ratings of discomfort.

Dependent variable	Model a: Retrospective discomfort	Model b: Willingness to do the task again
Peak-end discomfort	β’_peak_ = .65*	β’_peak_ = .001
	β’_end_ = .24*	β’_end_ = -.24*
	adj R^2^ = .73*	adj R^2^ = .05*
ΔR^2^ after adding an additional variable		
Average discomfort	ΔR^2^ = .038*	ΔR^2^ = .003
Initial discomfort	ΔR^2^ = .020*	ΔR^2^ = .001
Total discomfort	ΔR^2^ = .005	ΔR^2^ = .002
Duration	ΔR^2^ = .002	ΔR^2^ = .001
Performance accuracy	ΔR^2^ <.001	ΔR^2^ = .007
Perceived performance	ΔR^2^ = .011*	ΔR^2^ = .081*

*p ≤ .003;

The significance level was corrected for multiple comparisons using Bonferroni correction (α = .05/18 = .003).

**Table 5:** Hierarchical regression analysis predicting retrospective effort and the willingness to do the task again from real-time ratings of effort.

Dependent variable	Model c: Retrospective effort	Model d: Willingness to do the task again
Peak-end effort	β’_peak_: .49*	β’_peak_: -.03
	β’_end_: .36*	β’_end_: -.06
	adj R^2^ = .58*	adj R^2^ = .002
Adding an additional variable		
Average effort	ΔR^2^ = .034*	ΔR^2^ = .001
Initial effort	ΔR^2^ = .013*	ΔR^2^ = <.001
Total effort	ΔR^2^ = .007	ΔR^2^ = .003
Duration	ΔR^2^ = <.001	ΔR^2^ = .001
Performance accuracy	ΔR^2^ = .001	ΔR^2^ = .018
Perceived performance	ΔR^2^ = .006	ΔR^2^ = .110*

*p ≤ .003;

The significance level was corrected for multiple comparisons using Bonferroni correction (α = .05/18 = .003).

As indicated in Table 4, for Model a, real-time peak and end discomfort accounted for a significant amount of variance in retrospective discomfort (73%). Inspection of the standardized beta weights revealed that peak (β’ = .65) and end (β’ = .24) both predicted significant non-redundant variance in retrospective discomfort. Moreover, including real-time average discomfort (additional 3.8%), initial discomfort (additional 2%) and perceived performance (additional 1.1%) significantly improved model fit when predicting retrospective discomfort. For Model b, peak and end discomfort accounted for a significant amount of variance in willingness to do the task again (5%), while the standardized beta weights revealed that end discomfort (β’ = -.24) was the significant predictor. Moreover, including perceived performance (additional 8.1%) significantly improved model fit when predicting willingness to do the task again.

We conducted an additional analysis to predict retrospective discomfort when average real-time discomfort was entered first into the model, followed by peak and end real-time discomfort. When we did this, peak and end real-time discomfort continued to explain significant variance in the model (Δ*R*^2^ = .024 for the additional variance explained by peak and end real-time ratings over above the average, *p* < .001). This indicates that average, peak and end real-time ratings are not redundant with one another in terms of their ability to predict remembered discomfort. We obtained the same findings when initial real-time discomfort (Δ*R*^2^ = .26, *p* < .001) and perceived performance (Δ*R*^2^ = .62, *p* < .001) were entered into the model before peak and end real-time discomfort. In addition, we entered end real-time discomfort after perceived performance and found that end real-time discomfort continued to explain significant variance in willingness to do the task again (Δ*R*^2^ = .02, *p* < .002).

We conducted parallel regression analyses to determine whether real-time effort ratings would predict retrospective effort and willingness to do task again. As indicated in Table 5, for Model c, real-time peak and end effort accounted for a significant amount of variance in retrospective effort (58%). The standardized beta weights revealed that peak (β’ = .49) and end (β’ = .36) both predicted significant non-redundant variance in retrospective effort. Moreover, including real-time average effort (additional 3.4%) and initial effort (additional 1.3%) significantly improved model fit. For Model d, real-time peak and end effort did not predict willingness to do the task again, but instead perceived task performance (11%) accounted for a significant amount of variance in the willingness to do the task again.

We conducted an additional analysis to predict retrospective effort when average real-time effort was entered first into the model, followed by peak and end real-time effort. When we did this, peak and end real-time effort continued to explain significant variance in the model (Δ*R*^2^ = .03 for the additional variance explained by peak and end over above the average, *p* < .001). This indicates that average, peak and end real-time effort are not redundant with one another in terms of their ability to predict remembered effort. We obtained the same findings when initial effort (Δ*R*^2^ = .26, *p* < .001) was entered into the model before peak and end real-time effort.

## Discussion

### Subjective mental effort and discomfort

Participants reported that the working memory task required a large amount of mental effort and induced a large amount of discomfort. Moreover, ratings of real-time effort required and discomfort were highly correlated, and changes in ratings of real-time mental effort required were correlated with changes in real-time discomfort. Taken together these results are consistent with the oft asserted, but rarely empirically tested, claim that that the subjective experience of effort is unpleasant and aversive [11, 27–29]. Indeed, it would appear that most approaches to mental effort assume mental effort is *inherently* aversive. Although strongly correlated, the present findings suggest that subjective effort and discomfort are not identical, or two sides of the same coin. For example, changes in effort and changes in discomfort were significantly correlated at .46, however, this correlation leaves considerable variance unexplained. Further, real-time discomfort predicted willingness to repeat the task, but real-time effort did not predict willingness to repeat the task. In addition, our analysis of slopes of effort and discomfort show that ratings of effort tended to decrease over the course of the task and ratings of discomfort tended to increase over the course of the task. These findings suggest the simple point that it may not be valid to equate subjective mental effort and discomfort. Treating subjective effort required and discomfort as distinct, but correlated, opens up the possibility of questions such as what moderates or mediates the relation between effort and discomfort and what dynamic causal relation might they have over time and the possibility of individual differences in this relationship. For example, other work in our lab has shown that the correlation between real-time effort required and discomfort is significantly larger for those at risk for ADHD compared to those who are not at risk [30]. Further, conceptualizing mental effort required and discomfort as distinct phenomenological experiences provides another lens through which to examine existing theories of the psychological mechanisms underlying the experience of mental effort. For example, Kurzban et al. [11] argue that subjective mental effort is the conscious output of an opportunity cost / benefit analysis of continuing to perform the task at hand. They further suggest that because subjective mental effort is inherently aversive it motivates a discontinuation of the task at hand so that mental resources are not squandered on activities that offer little benefit. In response to this proposal Kool and Botvinick [11, 31] posit a simpler model in which the exertion of cognitive control is intrinsically costly, effortful and aversive. If indeed subjective mental effort and discomfort are distinct then these models would need to be revised in order to articulate specific relations (and possible dissociations) between the theorized underlying causal mechanism and subjective effort on the one hand and discomfort on the other hand. Clearly, further work needs to be done to fully establish the virtue and possibility of conceptually and psychometrically distinguishing between subjective mental effort and discomfort; but at the very least the results described here suggest it is unwise to simply assume they are one and the same thing.

### Retrospective memories of a cognitively demanding task

It is important to note that undergoing a colonoscopy is profoundly different from completing a cognitively demanding task. However, in at least one way they are similar. How a patient remembers the total pain of a colonoscopy [19] is similar to how a participant recalls the total mental effort required and total discomfort of a cognitively demanding task. Namely, in both cases, when people are asked to provide retrospective ratings, they do not sum up or aggregate real-time experiences but instead base their retrospective judgments on the peak levels (i.e. most intense) of pain, mental effort and discomfort and the final levels of pain, mental effort and discomfort. Consistent with the research on pain [19, 32] we also found that average real-time effort required and average real-time discomfort predicted unique variance in remembered ratings of total effort and remembered ratings of total discomfort respectively. Importantly though, just like the research on pain [19], average, peak and end ratings of discomfort and effort each accounted for unique variance in retrospective judgments, not just average effort alone. Moreover, total real-time ratings did not predict retrospective ratings. These findings show that remembered total pain, effort and discomfort is not an accurate reflection of the real-time total experience. Both with a colonoscopy [19, 32] and with a cognitively demanding task [16, 17], participants seem to neglect the duration of the ordeal when forming retrospective memories. The peak-end rule has been demonstrated in a large variety of painful procedures [15, 19, 33, 34], and in positive or negative affective events including the experience of exercise [35, 36], and assessment of life quality [37]. Here we extend these findings using parallel methods into the realm of cognitively demanding tasks.

Unlike Redelmeier and Kahneman (but consistent with Finn and Miele [18] and Redelmeier et al. [32]), we found that initial levels of discomfort predicted retrospective discomfort over and above peak and end real-time discomfort. Several methodological factors could account for this discrepant finding. In our study, initial ratings by our participants were more intense (i.e. initial real-time ratings in our study were approximately 4/7 whereas in Redelmeier and Kahneman initial real-time ratings were 3/10) and this may have made them more salient. In addition, participants in Redelmeier and Kahneman’s study made their retrospective judgments within an hour of finishing the procedure, whereas in our study retrospective judgments were made within minutes of finishing the cognitively demanding task. Perhaps the initial real-time ratings become less influential after the passage of time. Future research could examine if the role of initial ratings varies as a function of intensity and delay [33, 38] or subjective experiences of time [39]. Alternatively, it is entirely possible that retrospective memories of cognitively demanding tasks differ from retrospective memories of painful procedures with regard to the influence of initial ratings. Further research is necessary to disentangle these various possibilities, especially given the inconsistent findings within the pain literature. Zauberman, Diehl, and Ariely [40] argue that the impact of initial real-time experiences on retrospective evaluations varies depending on the kind of retrospective evaluations, with ‘informational evaluations’ being more related to initial experiences and ‘hedonic evaluations’ being more related to final experiences and changes in experience over time. Future research on retrospective evaluations of cognitively demanding tasks would benefit from systematically varying the kinds of evaluations participants make in order to see if this distinction between informational and hedonic evaluations holds for cognitively demanding tasks.

We found that peak (i.e. most intense) levels of real-time effort required and discomfort played a significant and unique role in predicting the retrospective evaluation of total effort required and discomfort. Finn and Miele [18] did not examine the impact of most intense moments of effort but rather they explored the impact of least intense moments of effort (which, somewhat confusingly they referred to as ‘peaks’). Therefore, in this regard, our findings are not in direct contradiction to the findings of Finn and Miele. Finn and Miele concluded that cognitively demanding tasks that end or begin easily are equally preferred and are actually more preferred than tasks that have relatively easy items embedded in the middle. Thus, they concluded that primacy and recency may be the key driver of retrospective judgments rather than endings and moments of relative ease. Several methodological factors could account for this different finding. First, as mentioned already our peak reflected moments of most intense effort and discomfort, whereas for Finn and Miele ‘peak’ reflected moments of least effort and discomfort. Therefore, it would be important to systematically explore the role of both maximum and minimum levels of real-time effort and discomfort in future research. Second, we varied peak (i.e. most intense moments) independently of initial and final levels of effort, whereas for Finn and Miele least effortful moments always and only occurred at the beginning, middle or end of the task. Thus, our design allowed us to explore the contributions of moments of intense effort and discomfort independently from the beginnings and endings in the prediction of retrospective memories. We intentionally designed our task so that ease/difficulty of the items was not tied to the beginning or end of our task, which differs from other experimental designs and may account for differences in findings (such as Tully & Meyvis [41]).

### Prospective willingness to repeat cognitively demanding tasks

As mentioned at the start of the discussion, we found that ratings of real-time effort and discomfort diverged in terms of their relation with willingness to repeat the working memory task. That is, we failed to find any evidence that ratings of real-time effort predicted willingness to repeat the task whereas end levels of real-time discomfort did predict willingness to do the working memory task again. While the null finding for mental effort required does not allow us to draw strong conclusions it would appear that real-time discomfort is related to willingness to repeat the task. This finding suggests that it is not the subjective experience of effort per se, but rather the subjective experience of discomfort during a cognitively demanding task that determines choices and preferences for subsequent behaviour.

Finn and Meile [18] found that longer cognitively demanding tasks (thus, with overall greater total discomfort) that ended or began on an easier note were chosen to be done again more often than shorter tasks (thus, with overall less total discomfort) that did not have easier beginnings or endings. In the present study, individual differences in reported subjective experience, as opposed to experimental task manipulations, were used to predict willingness to do the task again. Thus, our findings are based on a distinct methodology compared to Finn and Meile [18].

Much research still needs to be done to fully understand what real-time subjective experiences during a cognitively demanding task best predict willingness to repeat the task. Given learning contexts typically involve doing similar tasks repeatedly over time and require full engagement for maximal learning and task completion, resolving this question is critically important. As it stands now, learning contexts are typically structured in such a way as to begin more easily and terminate when the student experiences more difficulty. This structure may not be optimal for shaping positive memories of learning and for facilitating subsequent engagement (see [17] for an elaboration of these ideas). Thus far, all of the existing findings converge on the idea that ending on an easier note would facilitate greater willingness to repeat the task in the future.

### The impact of actual and perceived performance on future behaviour

Completing a cognitively demanding task, unlike undergoing a colonoscopy, involves the possibility of performing well or poorly. Intuitively it would seem plausible that how well an individual does on a task will influence his or her willingness to do the task again. Indeed, empirical evidence bears out this intuition. For instance Finn & Metcalfe [20, 21] have shown that learners track their performance and use this information to make judgments about learning. Moreover, Ariel et al. [22] have shown that learners chose to study material that will maximize their future performance. Our findings point to an important difference between actual and perceived performance. That is, how well participants thought they did after completing the task was positively correlated with willingness to do the task again, whereas actual performance was unrelated to willingness to repeat the task. This finding highlights the importance of asking participants about their perception of how well they are doing rather than simply relying on measures of actual accuracy. For some cognitively demanding tasks, such as the working memory task we used here, participants may have limited ability to judge how well they are actually doing, and in such circumstances it makes sense that perceived performance would be more robustly related to willingness to repeat the task.

In order to demonstrate a particular impact of real-time discomfort and effort on willingness to repeat a cognitively demanding task, it is necessary to disentangle the confound of performance. For example, Finn [16] utilized a design whereby the short task that ended with more difficult items yielded better performance than the longer task that ended with easier items. Thus, this study design ensured that participant’s task preferences were not determined by performance. Indeed, it was even more striking that participants preferred the task with higher levels of total discomfort and worse actual performance. Unfortunately, the Finn & Miele [18] design did not have this same control and, thus, confounded performance with task difficulty. In the present study, we were able to examine the independent effects of subjective effort, discomfort and perceived performance. We found that final real-time discomfort and retrospective perceived performance, but not actual performance or real-time effort provided unique, significant ability to predict willingness to do the cognitively demanding task again. Thus, taken together, it would appear that how uncomfortable a cognitively demanding task is when it ends and how well a person thinks they did on the task once it is completed both uniquely contribute to participants’ willingness to do the task again.

Previous work has shown that participants judgment of performance was found to be higher when easier math items were presented at the beginning compared to the end of a task [18]. Judgments of performance may be importantly shaped by the characteristics of a cognitively task, which may impact a participants’ willingness to complete further effortful tasks. This raises interesting implications for instructional design based on these findings. For example, beginning or ending on a high note and considering the most effortful moment may have significant effects on students’ self-regulation of learning. Further research will be needed to determine whether such effects lead to more engagement or even under-preparation by students [42]. For example, the work by Koriat et al. [2] demonstrates that students’ judgments of learning depend on the attributions they make about the experience of mental effort. The meaning given to the experience of mental effort as well as individual differences and environmental determinants of such meaning are fruitful themes for future inquiry.

### Limitations of present work and future directions

The conclusions reached based on the present findings should be tempered in light of three key limitations. Although, the present project is the first to vary duration beyond dichotomous long versus short conditions when investigating the determinants of memory for cognitively demanding tasks, our manipulation of ‘duration’ still did not span a very large range and possessed somewhat limited variability (i.e. five levels) for our analyses related to ‘duration neglect’. Interesting, in the Finn [16] and Finn and Meile [18] studies participants thought the longer condition that ended on an easier note was actually shorter. This raises the intriguing possibility that like our findings on actual versus perceived performance it may be useful to distinguish between actual and perceived duration in future research on duration neglect for cognitively demanding tasks. Second, given we employed a challenging working memory task to engender mental effort, participants’ ratings of mental effort started high and decreased significantly over the course of the task. This is important to take into account when considering the generalizability of our findings to other cognitively demanding tasks. Indeed, pilot work in our lab using a vigilance task to induce mental effort created a situation where real-time mental effort scores started low and increased significantly over time. Previous work by Ariely [43] examining how the rate and pattern of real-time pain ratings impact retrospective pain evaluations serves as good model for subsequent work examining the impact of dynamic changes in real-time mental effort. Third, in our regression models predicting willingness to do the task again, the overall R-squared was relatively small indicating that perceived performance and real-time measures of effort and discomfort were not strong predictors of willingness to do the task again. Future research may consider other variables that might determine willingness to do a mentally effortful task such as trait dispositional factors like the need for cognition [44] or attributions regarding the experience of mental effort [2]. On the other hand, future research could include different kinds of questions to assess behavioural intentions and desires, such as questions that will not be influenced by social desirability. In the present work, simply asking participants if they are willing to repeat a mentally effortful task may be subject to social desirability biases and thus not a sensitive measure of unconstrained behaviour intentions.

## Conclusions

The present project was predicated on the notion that it is viable and useful to study mental effort from the perspective of subjective self-report, here we come full circle and end on a discussion of this perspective. Rather than relying on ‘objective’ task difficulty [45] or performance [46, 47] as proxies for mental effort, we directly asked participants how effortful they found a working memory task. Moreover, rather than theoretically proposing [11, 48] or inferring the aversiveness of mental effort from task avoidance or preference [10], we directly correlated self-reported mental effort and discomfort. Further, rather than defining our independent variables in terms of objective task differences [47], we used subjective self-report as the key independent variables in our experimental design. On this point, it is important to note Ariely’s [43] caution that the act of providing real-time ratings may skew retrospective judgments to be more consistent with average real-time ratings than would be expected based on the impact of patterns of stimulus intensity and duration on retrospective judgments when participants do not provide real-time ratings. On the one hand, this might be interpreted as meaning that providing real-time ratings creates a bias. On the other hand, this might be interpreted as meaning that providing real-time ratings changes the “in the moment” experience. We agree that it is important to understand and minimize any potential impact of unintended experimental procedures on our investigations; we also assert, however, that self-report is the only way to directly and accurately assess subjective experience. Thus, we are faced with some tricky challenges related to experimental design and interpretation moving forward. In our view, these challenges reflect an acknowledgement of the value of studying the subjective experience of mental effort. In concluding we propose it is useful and possible to ground our definitions of mental effort in subjective experience [49], as has been done in the pain literature. Indeed, Westbrook and Braver [12] has recently made a similar argument: “Cognitive effort is a subjective, psychological phenomenon. It may covary with objective dimensions of task demands or incentive magnitude, but it cannot be described purely in these objective terms.” We view our present contribution as part of a growing focus on the subjectivity of mental effort.
